# HCV spread among female incarcerated population and treatment pathways to viral elimination in Italian prison settings: clinical perspectives and medico legal aspects

**DOI:** 10.1186/s12879-022-07565-2

**Published:** 2022-07-07

**Authors:** Vito Fiore, Elena Rastrelli, Giordano Madeddu, Roberto Ranieri, Andrea De Vito, Ruggero Giuliani, Giulio Di Mizio, Matteo Bolcato, Giuseppe De Matteis, Anna Maria Ialungo, Serena Dell’Isola, Giulio Starnini, Sergio Babudieri

**Affiliations:** 1 Infectious and Tropical Disease Clinic, Department of Medical, Surgical and Experimental Sciences, University of Sassari, Viale San Pietro 35/b, 07100 Sassari, Italy; 2 Medicina Protetta-Unit of Infectious Diseases, Belcolle Hospital, Viterbo, Italy; 3 Penitentiary Infectious Diseases Unit, A.O. Santi Paolo e Carlo, University of Milan, Milan, Italy; 4 Forensic Medicine, Department of Law, Magna Graecia, University of Catanzaro, Catanzaro, Italy; 5 Legal Medicine, University of Padua, 35121 Padova, Italy; 6 Health Protection for Adults and Youth Unit, Penitentiary Institute, Salerno, Italy

**Keywords:** Prison settings, Viral hepatitis, Incarcerated women, Micro-elimination, Clinical risk management, Medico legal aspects

## Abstract

**Background:**

Hepatitis C virus (HCV) infection is more frequent among incarcerated people than in general population. In the DAAs era, the short schedules and the low risk of adverse reactions, increased the number of HCV treatments. However, the most part of literature reports lack of incarcerated women inclusion in studies on field. Our aim is to assess the screening execution, HCV prevalence, and DAAs treatment among incarcerated women. A focused insight on quick vs standard diagnosis and staging approach will be also provided.

**Methods:**

Incarcerated women from 4 Italian regions’ penitentiary institutes were included. HCV screening was executed with HCV saliva test (QuickOral Test^®^) or phlebotomy. Stage of liver fibrosis was evaluated with FIB-4 value or fibroscan^®^, based on physicians’ decision. Treatment prescription followed national protocols.

**Results:**

We included 156 women, 89 (57%) were Italian, mean age was 41 ± 10 years, and 28 (17.9%) were people who inject drugs (PWIDs). Overall, the HCV seroprevalence was 20.5%. Being PWID and on opioid substitution therapy (OST) were significantly associated with serological status (*p*-value < 0.001). Of them, the 75.5% of patients had active infection, the most frequent genotype was 3a (50%). Among them, 4 (16.6%) and 6 (25%) had psychosis or alcohol abuse history. The 62.5%, 25% and 12.5% had low, intermediate, and advanced fibrosis, respectively. Out of the 24 HCV-RNA positive patients, the 75% underwent to DAAs treatment. The sustained virological response (SVR12) was achieved in 88.8% of cases. When evaluating the influence of quick diagnosis and staging methods vs standard phlebotomy and fibroscan^®^ on SVR12, FIB-4 use showed higher performance for retainment in treatment during prison staying (*p* = 0.015), while the use of quick saliva test had no influence on the outcome (*p* = 0.22).

**Conclusion:**

HCV seroprevalence and active infections are very high among incarcerated women. More tailored interventions should be focused on HCV diagnosis and treatment in female prison population. The use of quick staging methods (FIB-4) is useful to increase SVR12 achievement without delays caused by the fibroscan^®^ awaiting.

## Background

Hepatitis C virus (HCV) infection is more frequent among incarcerated people than general population [1]. The high-risk for HCV transmission makes incarcerated people a very important target population for creating specific HCV micro-elimination pathways [2]. In the direct acting antivirals (DAAs) era, the short schedules and the low risk of adverse reactions increased the number of treatments among HCV infected patients. For these reasons, numerous papers have been published on the feasibility and efficacy of HCV therapy in prison settings. Furthermore, literature on updates in HCV epidemiology and cascade of care in penitentiary settings is rising in the last years [3, 4].

Previous data from the Italian Ministry of Health highlighted how incarcerated women were twice more likely than incarcerated men and 14 times than the general population to have HCV infection [5].

Data from the Ministry of Justice show how incarceration among women is highly related to sex work and drugs, very often related to each other [6]. This makes female incarcerated population at high-risk for blood-borne viruses.

As per law from February 2020, the Italian Ministry of health allocated a specific fund for the introduction of free HCV screening for the unaware identification among specific subpopulations, such as incarcerated people and people who inject drugs (PWIDs) [7]. The objective is the HCV elimination, according to 2030 WHO targets, with a quick linkage to care in those specific settings.

Although the current national literature highlights how current micro-eradication strategies and new drugs could lead to similar results both in penitentiary settings and outside community, all available literature is concordant in defining incarcerated women as the harder-to-reach population [3, 8–10].

As of November 2020, out of 54,368 daily incarcerated people, 2303 (4.2%) were women, according to Ministry of Justice data [6].

Till now, there are no clear national data on screening execution, HCV prevalence reassessment, and DAAs treatment among incarcerated women.

Our study aims to describe the current HCV prevalence, active infection rates, clinical features, and the efficacy of direct acting antivirals (DAAs) among Italian incarcerated women. A focused insight on quick vs standard diagnosis and staging approach will be also provided.

## Patients and methods

### Patients’ definition

We consecutively enrolled incarcerated women from 4 Italian regions. The inclusion criteria were adult age (≥ 18 years old) and informed consent signing. Patients with previously known HCV screening were excluded. PWIDs, alcohol dependence, and psychiatric disorders were defined based on the dedicated psychiatric and dependency service, available in all Italian penitentiary institutes.

### The tests offer

HCV screening was executed with HCV saliva test (QuickOral Test^®^) or phlebotomy, based on availability or adherence to the SIMSPe micro-elimination project [3].

The screening tests were offered at prison admission (if quick tests, directly performed by the Specialists with dedicated nurses). Patients testing positive to QuickOral Test^®^ subsequently underwent blood testing for HCV-RNA (Siemens^®^), genotype, HIV, and HBV screenings. Patients performing phlebotomy had to wait the laboratory results before staging.

### Liver fibrosis evaluation

Liver fibrosis was evaluated with FIB-4 value [11] or fibroscan^®^, based on physicians’ decision. Fibroscan^®^ was programmed on territorial services. Patients with FIB-4 value > 3.25 and/or METAVIR F4 according to fibroscan^®^ underwent liver ultrasound to exclude hepatocellular carcinoma suspicion.

### Treatment prescription and delivery

Treatment prescription was telematical and regardless of liver stage of fibrosis, as per national protocols [12]. DAAs delivery (glecaprevir/pibrentasvir or sofosbuvir/velpatasvir) was within one week from staging tests availability, and administration was directly observed. Drug choice depended on patients’ characteristics, choice, or drug-drug interactions with chronic therapy.

### Linkage-to-care approach

There are no national indications regarding the patients’ length of stay in prison and DAAs prescription. However, to maximize the retainment in care, if the patients were next to release or transfer, they were linked by the Specialist to territorial services (referral centers/methadone clinics) or in the new penitentiary institute. The linkage-to-care was in charge of the Specialist who offered the screening and staging tests.

### Outcomes

Seroprevalence was based on HCV antibodies (HCV-Ab) detection. HCV active infection was defined by HCV-RNA positivity. End of treatment (EOT) was defined as treatment completion. The sustained virological response (SVR12) was defined as HCV-RNA negativity after 12 weeks from the EOT. Virological failure was defined as positive HCV-RNA either at EOT or at SVR12 control, and breakthrough as positive HCV-RNA after EOT negativity. Drop-outs were considered as: unplanned interruptions during treatment or not treatment start, due to unexpected release/transfer or patients’ decision.

### Sample size and statistical analysis

Based on female prison population [6], a prevalence up to 10.4% [3], a 0.05 precision and a confidence interval (CI) of 95%, the sample size needed was of 135 patients (CI specified limits 5.4–15.4%). Data distribution was evaluated with Kolgomorov-Smirnov test before analysis. Data were elaborated as numbers on total (percentages), means ± standard deviations (SD), and median (IQR). Categorical variables were evaluated with Chi-squared test or Fishers’ exact test, when appropriate. Logistic regression analysis was carried out to assess the relationship between HCV-Ab positivity/active HCV infection and epidemiological, demographic, and clinical variables, as well as to evaluate the relationship between quick testing and staging with SVR12. A two-tailed *p*-value less than 0.05 was considered statistically significant. Statistical computations were carried out with the statistical software STATA version 16 (StatsCorp, Texas, USA).

## Results

### HCV epidemiology

Out of the 190 Italian penitentiary institutes, 5 centers from four Italian regions participated, representing the 10% of female prison population.

From November 2020, 156 incarcerated women were consecutively enrolled. Of them, 89 (57%) were Italian, mean age was 41 ± 10 years, and 28 (17.9%) were PWIDs. Out of the 156 screened women, 134 (85.9%) were screened with HCV saliva test, while the others with phlebotomy.

Overall, 32 (20.5%) tested positive for HCV-Ab. Of them, 24 (75.5%) had positive HCV-RNA. PWIDs represented the most part of active infections (18; 75%).

The most frequent genotype was 3a (12; 50%). Regarding fibrosis evaluation, FIB-4 score was used in 15 (62.5%) cases. The remaining cases were staged with fibroscan^®^.

The majority of patients (15; 62.5%) had low fibrosis (FIB-4 score < 1.45; METAVIR F0-F1 according to fibroscan^®^). Among them, 4 (16.6%) and 6 (25%) had psychosis or alcohol abuse history. Baseline characteristics of incarcerated women who tested positive for HCV-RNA have been reported in Table 1.

**Table 1:** Demographics and clinical characteristics of HCV infected women enrolled in our study

Variable		Results (n = 24)
Italian nationality, n (%)		17 (70.8)
Age, mean ± SD		41 ± 9.5
PWIDs, n (%)		18 (75)
On OST, n (%)		13/18 (72.2)
Psychosis, n (%)		4 (16.6)
Alcohol abuse, n (%)		6 (25)
HBsAg, n (%)		1 (4.2)
HBcAb, n%		4 (16.6)
HIV, n (%)		6 (25)
Median (IQR) HCV RNA value		608,500 (339,575–1,449,312)
Genotypes, n (%)	1a	10 (41.6)
	1b	1 (4.2)
	2	–
	3a	12 (50)
	4	1 (4.2)
Fibrosis according to fibroscan^®^ and FIB-4 value*, n (%)	Low	15 (62.5)
	Intermediate	6 (25)
	Advanced	3 (12.5)

*IQR* interquartile range, *PWIDs* people who inject drugs, *OST* opioid substitution therapy, *ART* anti-retroviral therapy. *Low = METAVIR F0-F1 or FIB-4 < 1.45; Intermediate = METAVIR F2-F3 or FIB-4 between 1.45 and 3.25; Advanced = METAVIR F4 or FIB-4 > 3.25

When evaluating the relationship between HCV-Ab positivity, epidemiological and clinical variables, the logistic regression highlighted a statistically significant association with being PWID and on OST (*p*-value < 0.001), as reported in Table 2. However, active infection was not influenced by these factors.

**Table 2:** Logistic regression to evaluate the relationship between clinical and epidemiological variables and serological status for HCV among incarcerated women included in our study

	Unadjusted		Adjusted	
	OR (95% CI)	*p*-value	OR (95% CI)	*p*-value
Italian nationality	3.3 (1.3–8.3)	0.009	2.2 (0.6–7.9)	0.21
PWID	43.2 (14.3–131.2)	< 0.001	20.4 (5.4–76.8)	< 0.001
OST	139.4 (17.3–1123.4)	< 0.001	47.9 (4.6–495.2)	< 0.001
HIV	3.0 (1.0–8.8)	0.042	3.4 (0.9–12.9)	0.07

*PWID* person who inject drugs, *OST* opioid substitution therapy

When considering patients who tested HCV-Ab positive and HCV-RNA negative (8; 22.2%), 6 (75%) were previously treated with DAAs, and 2 (25%) had spontaneous eradication.

### HCV infection among PWIDs, level of awareness, and previous treatments

Evaluation of awareness on serological status did not show significant differences between PWIDs and non-PWIDs [PWIDs vs non-PWIDs = 14 vs 9 (*p*- value = 0.21)], as well as for active infection (*p*-value = 0.07). No significant differences in awareness were found also when considering only active infections (*p* = 0.06). Furthermore, no significant differences were found between PWIDs and non-PWIDs regarding previous DAAs treatments [PWIDs vs non-PWIDs = 4 vs 2 (*p*- value = 0.43)].

### HCV treatment and outcomes

**Fig. 1:**
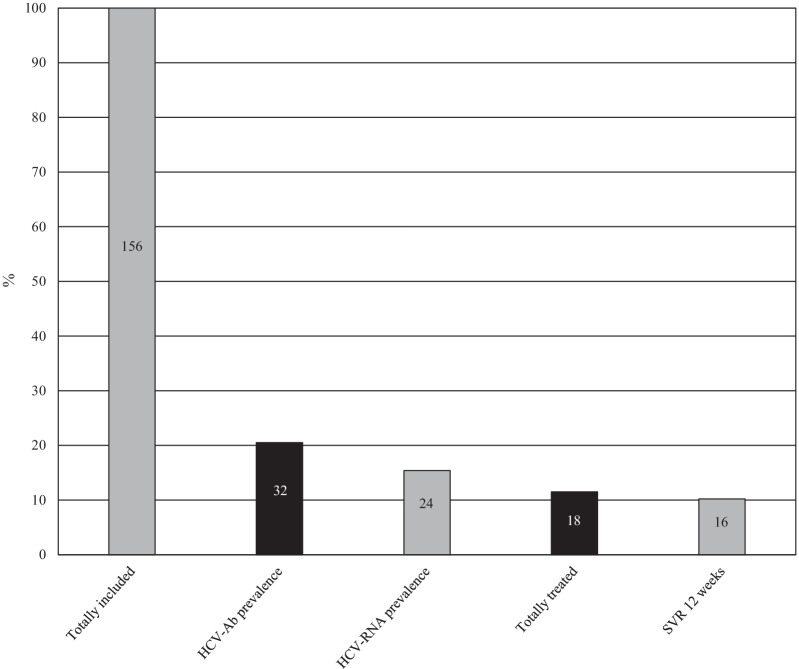
HCV cascade of care among 156 incarcerated women included in our study

Out of the 24 incarcerated women who tested HCV-RNA positive, 18 (75%) underwent to DAAs treatment. Prescribed drug schedules were 8-week therapy with glecaprevir/pibrentasvir and 12-week sofosbuvir/velpatasvir schedule in 11 (61.1%) and 7 (38.9%) cases, respectively. EOT was reached in 16 (88.8%) cases, and all of them achieved the SVR12. The remaining patients dropped out due to unexpected release to freedom (1; 5.6%) or transfer to another prison (1; 5.6%). The six remaining patients (25%) were released immediately before DAAs prescription but were linked to care in territorial services. HCV cascade of care has been reported in Fig. 1.

### Quick vs standard approach

When analyzing all the drop-outs (both unplanned interruptions and drop-out after prescription), FIB-4 use showed higher performance for retainment in treatment during prison staying (p = 0.015), while the use of quick saliva test had no influences on the outcome (*p* = 0.22). Logistic regression aiming to evaluate the relationship between SVR12 and quick screening and staging methods has been reported in Table 3.

**Table 3:** Logistic regression carried out to assess the relationship between SVR12 and quick screening and staging methods among incarcerated women with HCV infection included in our study

	Unadjusted		Adjusted	
	OR (95% CI)	*p*-value	OR (95% CI)	*p*-value
FIB-4	13 (1.7–99.4)	0.013	19.5 (1.8–214.6)	0.015
Quick saliva test	2.2 (0.3850–12.6)	0.37	4.5 (0.4–51.6)	0.22

## Discussion

This is the largest HCV survey among incarcerated women ever conducted in Italy. Incarcerated women are one of the harder-to-reach populations. In Italian penitentiary institutes, < 5% of incarcerated people are female. The limited women inclusion is one of the biggest limitations in the most part of the studies on prison settings [3, 8–10]. As consequence, the Specialist activity to reach them out should be pro-active.

We found a HCV seroprevalence of 20.5%, which is slightly higher than the prevalence reported in the more recent literature in field. In fact, the most recent studies show a HCV-Ab prevalence < 15% [3, 4]. Moreover, the most part of included patients were surprisingly viremic. This highlights the necessity of better targeted interventions on female prison population.

Mainly, patients had a low liver fibrosis, datum concordant with the low mean age of included women, and with the previous literature in field [3, 4].

As part of our results, HCV-Ab positivity was related to being PWID and OST, but active infection had not relationships with demographical and clinical features. Furthermore, level of awareness was not statistically different between PWIDs and non-PWIDs. This is divergent from our previous data [3]. However, the sample of both HCV-Ab positive and chronically infected patients was small, and this could have influenced the analysis.

High SVR12 rates were reached (~ 90%). The patients undergoing to quick staging methods (FIB-4) were more likely to achieve SVR12 than people undergoing fibroscan^®^. This could be related to the reduced delays when compared with fibroscan^®^ awaiting, with less possibility of unplanned interruptions due to unexpected prison release or transfer. Surprisingly, there was no difference between saliva quick test execution and phlebotomy. However, data came from penitentiary institutes where the HCV testing is offered in routine clinical practice at prison admission. This can represent a bias, making this result not necessarily applicable to the whole national context. In fact, the better performance of an approach based on a step-by-step model of quick screening and fast-track staging and treatment in prison micro-environments has been well described by literature [3]. HCV point-of-care testing with quick tests has been widely recognized as a feasible screening method, which gives the possibility of HCV diagnosis among underserved populations, such as incarcerated people [13–15]. The use of quick applicable scores (FIB-4) instead of instrumental staging, was in line with these data.

Given the difference in terms of SVR12, it seems the accessibility to fibroscan^®^ has been overcome by the other indirect staging methods, with a quicker DAAs prescription. Despite this, Italian penitentiary institutes are still not homogeneous in the diagnostic and therapeutic paths. Prison settings are an extraordinary occasion for an extensive approach to HCV fast-track test, staging, and treatment. Targeted interventions should be improved for these settings [16]. Furthermore, the possibility of a directly observed treatment is an added value for creating better micro-elimination pathways [17–19]. A high advantage was represented by the linkage-to-care on territorial services. This kind of approach is increasingly being used nationwide, allowing to maintain in care the patients, even after release.

In conclusion, to increase HCV prevention and a quick test, staging and treatment strategy is highly beneficial in prison settings, as well described by literature [20, 21]. Incarcerated women are a minority of Italian prison settings populations, with higher difficulties to reach them without specific programs. A gender specific network would be useful to increase the retainment in care of this subgroup.

### Clinical risk management and medicolegal aspects.

The Italian law n. 24 of 2017 recognizes the health of citizens as a constitutionally guaranteed right, also through the objective the safety of care to be guaranteed in every health activity [22]. This right is ensured through the implementation of clinical risk management and prevention activities in every healthcare setting, either hospital or territorial.

The penitentiary administration has custody of people deprived of personal liberty. As consequence, it should provide health services thorough the National healthcare system. Moreover, the rights of restricted citizens cannot be less considered than those of free citizens. A general analysis of the system makes it possible to appreciate how the same health management problems exist in the prison world as global health systems for free citizens, with further difficulties represented by the criticality of identification, acquisition and interpretation of the performance and safety indicators of the patient [23].

The diagnostic-therapeutic pathways guaranteed inside the penitentiary system may encounter obstacles. For this reason, optimal counselling on blood-borne viruses and the creation of simplified microelimination pathways is fundamental.

In conclusion, our survey shows how the opportunity to test and treat viral hepatitis in prison settings is useful not only from a clinical and diagnostic point of view, but also in medical-legal terms and—strategically—in terms of prevention risk.

### Limitations of the study

Some limitations should be addressed regarding our study. First of all, our data came only from voluntary self-included penitentiary institutes, which represent a minority of the whole female incarcerated population. For this reason, our results may not reflect the whole national situation. Furthermore, screening and staging methods were not homogeneous in all centers. This may represent an important bias of the study. A limited number of clinical variables were included in our analysis, as well as a small patients’ sample. More institutional coordination is needed in order to create common point of view and strategies. Regarding the screened and staged patients who did not start DAAs during prison stay, they were linked to territorial services, but we do not have data on their treatment. A better national network development, involving Ministry of Justice, Health, methadone clinics, and General Practitioners would allow to better evaluate the treatment success after release.

## Data Availability

The datasets used and/or analysed during the current study are available from the corresponding author on reasonable request.
